# Navigating the Challenges of Factor X Deficiency: A Case Study

**DOI:** 10.7759/cureus.65084

**Published:** 2024-07-22

**Authors:** Gaurav A Chaudhary, Harin M Bhavsar, Madhulika L Mahashabde

**Affiliations:** 1 General Medicine, Dr. D. Y. Patil Medical College, Hospital and Research Centre, Pune, IND

**Keywords:** factor x deficiency, bleeding risk, autosomal recessive disorders, hemophilia-a, hemophilia b, pt-inr

## Abstract

The clotting pathway involves intrinsic and extrinsic pathways converging into a common pathway. These pathways require activated factors that sequentially convert prothrombin to thrombin, which then converts fibrinogen to fibrin, forming a stable clot. Clotting factor deficiency impairs this cascade leading to excessive bleeding or bruising due to insufficient clot formation. Here, we present the case of a 47-year-old female who initially complained of epigastric pain. By the third day of admission, she experienced four to five episodes of bleeding gums, resulting in a blood loss of approximately 300 mL. The patient exhibited abnormal prothrombin time (PT) and international normalized ratio (INR) values, leading to a diagnosis of Factor X (FX) deficiency upon further evaluation. This case report emphasizes the need to diagnose coagulopathies such as FX deficiency and how early diagnosis will help not only in patient care and management but also in screening family members who may be affected.

## Introduction

The coagulation cascade consists of two pathways, intrinsic and extrinsic, which converge at the activation of Factor X (FX), leading to the common pathway where prothrombin is converted to thrombin, resulting in blood clot formation. FX is a vitamin K-dependent factor necessary for the coagulation cascade of the common pathway, which plays a vital role in fibrin formation. FX deficiency is a rare, recessively inherited bleeding disorder representing 10% of all rare bleeding diseases and affecting one in a million people, less than 5% of whom are likely to suffer from hemophilia A and B [1]. Laboratory investigations of FX deficiency include activated partial thromboplastin time (aPTT) and prothrombin time (PT). People with FX deficiency have prolonged aPTT and PT and a higher international normalized ratio (INR). FX deficiency is associated with malignancies, connective tissue diseases, liver disease, acute myeloid leukemia, infection, primary amyloidosis, and vitamin K deficiency [2,3]. While hereditary FX deficiency occurs in one out of a million people, it may involve more than a hundred genes because it is passed from the parents to the child in an autosomal recessive manner. The FX antigen activity in the plasma can also be used to categorize the condition into two groups: the first having less factor activity with a lower antigen level and the second having reduced factor activity with a normal antigen level.

Moreover, FX deficiency can be classified into three categories based on the degree of factor activity: mild, moderate, and severe. Mild FX deficiency may be screened during routine screening or in relatives of patients with FX deficiency [4,5]. The disease manifests in a moderate form in 6-10% of cases and is associated with nosebleeds or heavy menstruation. The moderate form of the disease occurs when the level of FX is 1-5%. Patients who are moderately afflicted with the condition can only be identified following surgery, trauma, or menstruation. Less than 1% of the population has an activity level associated with the severe form of the disease. Severe FX deficiency can occur during the neonatal period and may lead to bleeding from the umbilical stump, vitamin K deficiency, gastrointestinal bleeding, or intracranial hemorrhage. Bleeding risk is high in the moderate-to-severe form of the disease, with intravascular bleeding mimicking arthropathy, severe hemophilia A, or hemophilia B.

## Case presentation

A 47-year-old female, a housewife from a lower socio-economic background, presented with complaints of epigastric pain and dyspepsia for five days. The pain was an intermittent, non-radiating, burning type, and aggravated after taking food. There was no history of fever, vomiting, loose stools, or weight loss. Nor was there any history of chest pain, sweating, or palpitation on exertion. On presentation, her pulse rate was 90 beats/min; her blood pressure was 110/70 mmHg in the supine position. On general examination, pallor was found to be present. No icterus, cyanosis, clubbing, edema, or lymphadenopathy were detected. The patient was conscious, cooperative, and oriented to the time, place, and person; thus, the systemic examination was normal. The chest X-ray was normal, and the ECG showed normal sinus rhythm. The echocardiography was normal. Ultrasonography of the abdomen pelvis suggested grade I fatty liver. On lab investigation, the hemogram showed mild microcytic hypochromic anemia. The patient’s liver function test and urine routine microscopy were normal. Upper gastrointestinal endoscopy was done, which showed a rapid urease test positive suggestive of *Helicobacter pylori* infection.

On the third day, the patient had a sudden onset of four to five episodes of bleeding gums leading to blood loss of 300 mL. No similar episodes had occurred with her in the past. There was no history of hematemesis, melena, hemoptysis, petechiae, ecchymosis, or trauma. There was no associated history of fever, rash, joint pain, consumption of alcohol, tobacco, oral anticoagulants, or oral contraceptive pills. The patient also did not have any history of hypertension, diabetes, or liver disease. There was no significant family history. The patient had a deranged PT (29.4) and an INR (2.54). Bleeding time was 1 minute and 40 seconds, and clotting time was 4 minutes and 20 seconds. Since the patient had no bleeding from any other site, no rash, and no history of alcohol consumption, oral contraceptive pill use, or use of anticoagulants, factor assays were performed to investigate coagulopathies. Factor assays suggested FX deficiency. To treat the deranged PT and INR, the patient was given 12 units of fresh frozen plasma (FFP) for three days.

Due to the autosomal recessive pattern of the disease, the patient and her relatives were counseled on the hereditary nature of the disease and explained the need for FX deficiency evaluation of other family members. On further inquiry, it was found that the patient’s brother was also admitted with a history of hemoptysis and diagnosed with FX deficiency. The patient’s laboratory results on admission are shown in Table 1.

**Table 1:** Laboratory investigations SGOT and SGPT are enzymes that measure liver function. ALP is an enzyme that reflects bone and liver health. PT and INR are tests for blood clotting function, while aPTT assesses another aspect of clotting. Measurements like g/dL, mg/dL, µg/dL, and ng/mL are units of concentration. NPP and hpf are specific terms used in medical diagnostics. SGOT: serum glutamic oxaloacetic transaminase; SGPT: serum glutamic pyruvic transaminase; ALP: alanine phosphatase; PT: prothrombin time; INR: international normalized ratio; aPTT activated partial thromboplastin time; NPP: normal pooled plasma; hpf: high power field

Laboratory investigation	Reports	Reference range
Hemoglobin	8.0g/dL	13.2-16.6 g/dL
Total leucocyte count	7300/ µL	4,000-10,000 /µL
Platelet count	245000/ µL	1,50,000-4,10,000 /µL
Serum urea	18mg/dL	17-49 mg/dL
Serum creatinine	0.58 mg/dL	0.6-1.35 mg/dL
Total serum bilirubin	0.48 mg/dL	0.2-1.2 mg/dL
Direct bilirubin	0.23 mg/dL	0.5 mg/dL
SGOT	19 IU/L	8-48 IU/L
SGPT	15 IU/L	7-55 IU/L
ALP	54 IU/L	40-129 IU/L
Serum protein	6.9 g/dL	5.5-8 g/dL
Serum albumin	4.1 g/dL	3.5-5 g/dL
Albumin: globulin ratio	1.46	1.1-2.5
Serum iron	15 µg/dL	35-145 µg/dL
Total iron binding capacity	520 µg/dL	250-450 µg/dL
Transferrin saturation	2.88%	20-50%
D dimer	110 ng/mL	0-500 ng/mL
Fibrinogen	220 mg/dL	180-350 mg/dL
PT	29.4 secs	10.66-12.66
INR	2.54	<1.1
APTT	38.9 secs	23.83-31.68
Factor VII	100%	50-150% of NPP
Factor VIII	130%	50-150% of NPP
Factor IX	110%	50-150% of NPP
Factor X	1.9%	50-150% of NPP
Urine Microscopy		
RBCs	Absent	0-2 hpf
Pus cells	1-2	0-5 hpf
Epithelial	1-2	0-5 hpf

## Discussion

FX deficiency is a rare hereditary coagulopathy that affects the routine clotting process. This leads to episodes of bleeding, either spontaneously or after trauma or surgery, becoming longer than usual. Certain cases of FX deficiency involve normal levels of FX secretion, but it cannot function correctly; other cases involve no secretion of FX. Individuals with about 40% or more protein activity have mild disorders and are generally asymptomatic. Patients with 10-40% FX activity have moderate FX deficiency and present with complaints of bleeding manifestations such as epistaxis, hemoptysis, or menorrhagia. Patients with 10% or less FX activity, especially those with less than 1% FX, suffer from severe FX deficiency. The mode of inheritance for FX deficiency is autosomal recessive. A person with recessive genetic illnesses receives a mutated gene from each parent. The person then acts as a carrier of the disease if they have one normal gene and one mutated gene for the disease, but usually, they do not show symptoms. In the case of two carrier parents, the risk of them having an affected child is about 25%, a carrier child about 50%, and a normal child about 25%. The risk is the same for both sexes. A comprehensive clinical evaluation, a range of specialized tests, a complete patient and family history, and the identification of symptoms such as bleeding signs are necessary to diagnose FX deficiency. Screening coagulation tests, such as aPTT and PT, are required. Individuals deficient in FX have increased aPTT and PT. A FX assay will show decreased FX activity in afflicted people. FX deficiency can be diagnosed using molecular genetic testing. The FX gene variant can be found by molecular genetic testing, which is only offered as a diagnostic service at specialized facilities. Antifibrinolytic medications and specialized therapy are typically adequate for mild bleeding symptoms. FX replacement therapy, FFP, or plasma-derived prothrombin complex concentrates, which include a lot of activated vitamin K-dependent factors, can be used to treat more severe bleeding episodes [6-8].

## Conclusions

FX deficiency is a rare genetic disorder that impairs blood clotting leading to symptoms like easy bruising, frequent nosebleeds, bleeding gums, and heavy menstrual bleeding. The risk of severe bleeding necessitates early diagnosis and prompt treatment. Diagnosis of FX deficiency involves blood tests that measure clotting time and FX activity levels. Treatment options include plasma transfusions, prothrombin complex concentrates, or recombinant FX to manage and prevent bleeding episodes. Additionally, due to the autosomal recessive nature of the disease, family members should be screened. To treat FX deficiency, a team of professionals, including pediatricians, physicians, and hematologists, may need to work together. It is also crucial to provide psychosocial assistance for the entire family.
